# Why partner? Harnessing value from collaborative sustainable business models to restore coral reefs at scale

**DOI:** 10.1371/journal.pone.0315094

**Published:** 2024-12-16

**Authors:** Taryn M. Kong, Bruce Taylor, Victoria Graham

**Affiliations:** 1 Commonwealth Scientific and Industrial Research Organisation (CSIRO), Environment, Brisbane, QLD, Australia; 2 School of Geography, Planning, and Spatial Sciences, University of Tasmania, Hobart, TAS, Australia; 3 The Cairns Institute, James Cook University, Cairns, QLD, Australia; King Abdulaziz University, SAUDI ARABIA

## Abstract

Tropical coral reefs provide a wide range of ecosystem services that benefit millions worldwide. However, the current scale of coral reef restoration is a long way from matching the extent needed to protect coral reefs globally, and this implementation gap presents a complex challenge to overcome. Cross-sectoral collaborative sustainable business models (CSBMs) present an interesting opportunity to scale up coral restoration, though this area is yet to be explored in the literature. In this paper, we use the Reef Restoration and Adaptation Program in the Great Barrier Reef as a case study to examine potential collaborators, their roles, and what benefits motivate them to partner for scaling coral restoration. We identified a diverse range of potential collaborators from 10 sectors offering different combinations of physical, human and organisational capitals. Participants described nine roles they could play in such a partnership, and many of these roles relate to ecosystem growth scaling strategies. Benefits that motivate collaboration fall into seven categories: environmental benefit, business opportunity and value, employment opportunity, knowledge and technology, innovation, hope, and reputation. Our findings contribute to designing CSBMs for coral restoration by enriching our understanding of collaborators, value creation and their potential roles in alternative pathways to scale up coral restoration beyond reducing unit cost and increasing funding.

## 1 Introduction

Tropical coral reefs provide a wide range of ecosystem services that benefit millions of people worldwide [1]. Some of the most well-studied provisioning services include fisheries [2], cultural services include recreation and tourism [3], and regulating services include coastal protection [4] and nutrient processing [5]. Yet, few reefs have escaped exposure from local threats in the form of overfishing, catchment runoff and direct habitat destruction, and global threats in the form of increasing regional ocean temperatures and changes to the acidity of ocean waters [6–9]. Global coverage of living coral has declined by half since the 1950s, and at least 63% of coral-reef-associated biodiversity has declined [9]. The 2015–2018 global coral bleaching event affected 74% of reefs worldwide, with greater than 30% of coral cover lost on the Great Barrier Reef alone [10]. Trajectories for coral reefs under present greenhouse gas emission scenarios are dire, with 60% of all remaining coral reefs critically threatened and 98% exposed to environmental conditions above the current thresholds considered necessary to maintain ecosystem function as soon as 2030 [11].

Coral reef restoration is increasingly recommended as an active management strategy to address the deterioration of the state and expanse of coral reefs. In 2019, a report from the International Coral Reef Initiative (ICRI)’s ad-hoc committee on coral restoration revealed that 88% of ICRI countries are now using coral restoration as a tool to assist the recovery of coral reefs [12]. A global review found that coral restoration had been implemented in at least 56 countries as of 2020 [13], with most projects in low-income or developing nations. Coral reef restoration is now embedded in the Post-2020 Global Biodiversity Framework and signed by parties to the Convention on Biological Diversity (CBD) and resolutions from the United Nations Environment Assembly (UNEA Resolution 4/13).

For coral reefs, the term restoration is used to encompass restoration ("the process of assisting the recovery of an ecosystem that has been degraded, damaged, or destroyed…to return an ecosystem to its historical trajectory" [14]) and rehabilitation that does not aim to achieve pre-existing biotic conditions. The majority (60%) of coral restoration projects globally are short-term, lasting less than 18 months [13] and are small scale, with the median size of the restored area estimated 71 m^2^ [15] and 100 m^2^ [13]. Some literature also expands the purpose of coral restoration to include building reef resilience and adaptation to the impacts of global climate change [16, 17]. Novel interventions such as assisted genetics/evolution, assisted microbiome, and coral epigenetics to enhance the natural adaptive capacity of coral holobionts are being developed and tested for scalability to work at the reef dimension [18]. The gap between the scale of current coral restoration practices and what is needed to match the rate of degradation and extent of threats faced by coral reefs around the world is glaring [15, 19] and a complex challenge to overcome.

Scaling up coral restoration to enable a sustainable future for coral reefs in the face of climate change is a complex problem for multiple reasons. Knowledge gaps include incomplete cost modelling due to a lack of standardisation of restoration costs [20] and uncertainty in how to logistically scale restoration across entire reef systems. The estimated median cost per hectare varies greatly in studies–ranging from USD 50,000 to USD 1,000,000 [15, 21]. Taking the uncertainty of the large variation into account, restoring coral reefs is still seen as considerably more expensive than terrestrial restoration [15, 19], and the scale of coral restoration is currently constrained by substantial costs [20, 22]. Market mechanisms to generate revenue for restoration efforts are rare because reefs are a common-pool resource. A global survey shows that short-term grants, primarily from governments and the private sector, dominate the coral restoration funding landscape in the last 10–15 years [20]. Private sector players such as those in risk management have created innovative financing mechanisms in the form of reef insurance schemes or emergency management strategies [23], but this funding source is relatively minor compared to grants [20]. Factors such as limited market-based funding and uncertain cost modelling constraint the scalability of business models for coral reef restoration.

Collaborative sustainable business models (CSBM) have been suggested to bring together research on collaboration and sustainability business models to innovate solutions for complex sustainability challenges [e.g., 24–26]. Collaborative business models involve forging an alliance with partner organisations [27], and these organisations can be from the public, private and/or nonprofit sectors in cross-sectoral collaborations [24, 26]. A business model describes, as a system, how the pieces of an organisation fit together to create, deliver and capture values [28]. A sustainable business model adopts a more holistic perspective of business, broadening the understanding of value creation to include economic, social, and environmental benefits and customers to include beneficiaries or stakeholders of these values [e.g., 29, 30]. There is a myriad of examples in the energy, fashion, healthcare, food, construction, and hospitality industries globally, where sustainable business model principles are applied to achieve economic, environmental, and social goals simultaneously [31]. Coral restoration aligns with the ethos of a sustainable business model as it generates multiple financial and non-financial values for a wide range of beneficiaries. CSBMs provide a mechanism to address complex problems through collective perspective and actions, mobilising a broader base of resources while ensuring equitable sharing of costs and benefits [32].

While there is considerable literature on cross-sectoral collaborations, including those on coral restoration [e.g., 39–41], this literature does not study collaborations through a CSBM lens, or speak to the implications for CSBM development. A CSBM lens helps to unpack the business logic of a collaboration (cross-sectoral or not) to create, deliver and capture values. This paper addresses this knowledge gap by using a case study, the Reef Restoration and Adaptation Program in the Great Barrier Reef (see Section 3.1 for contextual background), to examine the following research questions:

- Who are potential collaborators in coral restoration CSBMs?
- What roles can these potential collaborators play in scaling CSBMs for coral restoration?
- What benefits or values would motivate these collaborators to partner?

The above research questions are deliberately exploratory and open to the latent potential that exists amongst diverse actors to participate in future restoration collaborations supported by CSBMs. Because of this exploratory intent we opted for an analytical approach that was not restricted to the use of a business model canvas [28] or that relied entirely on interviewees with existing engagement in CSBMs. However, when considering the implications of our analysis, we reflect on what our findings mean for developing scalable CSBMs. In particular we reflect on key concepts of collaborator resources and scaling strategies which we overview below. In doing so we offer a novel lens to the cross-sectoral collaboration literature addressing the complex challenge of large-scale coral restoration.

## 2 Theoretical background

### 2.1 Collaborator resources

Resources are a useful lens to study partnerships in collaborative business models [24, 25] because acquiring resources not available in-house is a crucial motivation for collaborating with external partners [33]. Such a perspective assumes that businesses are heterogeneous entities that achieve competitive advantage by possessing a bundle of tangible and intangible resources [34]. For example, acquired resources can help a business gain a competitive advantage by improving its strategic positions [24], managing risks and uncertainty [35], accessing valuable networks [36] and acquiring competencies it cannot develop [37] among others.

These resources can be categorised into four types of capitals: physical (e.g., technology, machines, infrastructure), human (e.g., experience, knowledge, skill, training), organizational (e.g., leadership, relationships, network, culture), and financial (e.g., debt, equity, earnings) [34, 38]. A collaborator’s competitive advantage reveals what resources it possesses. Such a classification framework can be used to examine how potential collaborators fit into a business model. The importance of understanding collaborators–who they are, what they can bring, and what motivates them–to business model development is reflected by the fact that partners are also a key element in a business model canvas [28]. The classification can also be used to identify resource gaps to scale up a business model and potential roles for partners to fill those gaps.

Coral restoration literature uses the term, stakeholders, to refer to local communities, charitable organisations, citizen scientists, tourism operators, coral reef managers, scientists and funders [13, 39–41]. Stakeholder roles usually involve providing funding, knowledge and know-how, buy-in and labour. In this sense, these stakeholders are potential partners in a CSBM. While collaborator resources offer a valuable analytical perspective, it has not previously been applied to understand stakeholders and how they may contribute to scaling up CSBMs for coral restoration.

### 2.2 Scaling strategies

In addition to gaining competitive advantages, businesses collaborate with other organisations to scale their operations or impacts [26, 35, 36]. For example, newcomers can benefit from collaborating with existing businesses to share their resources, access a customer base for the diffusion of a product or service, reach a new market segment, and gain intangible assets such as credibility, legitimacy and power [42, 43]–all are important to achieving scale [36].

A framework for scaling strategies can be used to understand collaborators’ roles in scaling operations or impacts. One group of scaling strategies seek to address sustainability challenges on a large scale by growing organisational size [44]. Organisational growth strategies encompass the expansion of products, services, interventions or programs to address the targeted sustainability problem, and the expansion of geographic coverage to reach larger numbers of beneficiaries [e.g., 45–47]. A second group of strategies indirectly addresses targeted sustainability problems on a large scale by growing or sustaining a supportive business ecosystem [44]. A business ecosystem perspective assumes that a set of attributes such as networks, investment capital, market infrastructure, and governance structure collectively create a supportive environment for a business to thrive [48, 49]. Ecosystem growth strategies encompass work relating to advocacy (e.g., influencing public policymakers, raising public awareness), building or supporting coalitions, building the legitimacy of organisations, establishing or nurturing a new industry, developing or sharing new infrastructure, financing, creating or disseminating knowledge, and training and advisory support [e.g., 29, 50–56].

The scaling strategy framework developed by Islam [44] provides a more holistic lens to examine collaborators’ roles in scaling than the typology proposed by de Man and Luvison [35], which focuses on more formalised collaborative business models. This typology categorises collaborative business models into sharing, specialisation and allocation, where collaborations are forged to achieve economy of scale, skill, and risk, respectively [35]. These three scaling strategies are more limited in scope in understanding how collaboration can influence the business ecosystem to support scaling as they tend to focus on the collaborating organisations. Scaling current coral restoration practices to the rate and extent needed globally fundamentally involves broader strategies addressing system-level attributes. In this way the framework developed by Islam [44] is particularly helpful for our analysis.

## 3 Methods

### 3.1 Case study context

This transdisciplinary research was undertaken as a project activity of the Reef Restoration and Adaptation Program (RRAP) to better understand perspectives and insights from organisations interested in collaborating on future coral restoration in the Great Barrier Reef (GBR). RRAP is a 5-year research program funded by the Australian Government’s Reef Trust Partnership with the Great Barrier Reef Foundation to create a suite of measures to help preserve, restore, and adapt the GBR in the face of climate change [57]. As the world’s largest coral reef system, it covers an area of 348,000 km^2^ –about the size of Italy. It is an integral part of Australia’s national identity holding deep cultural connections for over 70 of Australia’s First Nation communities living adjacent to it [58]. It contributes more than AUD 6 billion a year to the economy, and supports an estimated 64,000 jobs [59]. Its significance extends beyond the national border as one of the world’s seven natural wonders and a biodiversity hotspot.

Since 2016, the GBR has undergone five major bleaching events, with two of these events resulting in widespread coral mortality [60]. The cumulative threats of coral bleaching, outbreaks of the invasive crown-of-thorns starfish (*Acanthaster planci*) and tropical cyclones have led to 350 scientists from the Australian Institute of Marine Science, Commonwealth Scientific and Industrial Research Organisation, and several universities, to collaborate on developing, testing and implementing scalable interventions to protect the future of the GBR, such as coral spawn collection and larval reseeding ("larval reseeding" thereafter), propagation and resettlement of coral with improved traits ("coral propagation" thereafter), and cooling and shading.

This study, nested in the Stakeholder and Traditional Owner Engagement Sub-program of RRAP, has four phases: a) scoping and research design; b) situational analysis through interviews; c) deeper examination of approaches to addressing scaling challenges through case studies; and d) translation of case study learnings and insights to inform RRAP’s scaling strategy. The transdisciplinary approach referred to the study involving non-scientists to co-produce knowledge, and define specific research methods and questions in the third and fourth phases. This paper pertains to the findings from the second phase, where we conducted 33 semi-structured interviews with individuals from organisations interested in collaborating on future coral restoration in the GBR. The project was approved by the CSIRO Social Science and Human Research Ethics Committee (Application number: 182/21) in February 2022. A written free prior informed consent was received from each interview participant.

We provide further details on how the interview participants were identified and recruited, the interviews, and data analysis in the following section, 3.2.

### 3.2 Participants, interviews and data analysis

A snowball sampling technique was used to identify interview participants. Snowballing or chain referral is a commonly used sampling method in qualitative research to identify participants, where referrals to new potential participants are elicited from existing participants [61]. To start the snowballing, the project team tapped into the social networks of the RRAP researchers and management to identify a small number of initial contacts (seeds).

All 95 of the referred contacts, their sectors, and reasons for their referrals (if applicable) were recorded in a table. This information was used to prioritise which referrals to recruit to achieve a participant diversity that reflects the referred population. Eighty-seven of the 95 referrals were categorised into a specific sector, and eight were multi-stakeholder platforms or advisory groups representing multiple sectors. Fifty of the 95 referrals were invited based on a criterion to achieve as diverse representation as possible from the total referrals. We interviewed 33 participants between February and May 2022 –achieving a 66% response rate. The 33 interviews cover all 11 sectors of the 87 referrals (Table 1). Three participants were connectors to the 8 referred multi-stakeholder platforms and advisory groups. The relative distribution of participants across the sectors was even, except for government, non-for-profit (NFP), contractor and tourism, where there was a relatively higher recruitment. The relative distribution of the participants by sector mirrors that of the referrals, as shown by the small percentage difference in the last column of Table 1.

**Table 1:** Count of referred contacts and recruited participants by sector and relative percentages.

	A	%A = A_n_/ΣA	B	%B = B_n_/ΣB	%A-%B
Sector	Total referrals (count)	Relative proportion of referrals (%)	Participants (count)	Relative proportion of participants (%)	Difference in relative proportion (%)
**Aquarium**	7	8%	3	10%	-2%
**Charter yacht**	3	3%	1	3%	0%
**Citizen science**	5	6%	1	3%	3%
**Contractor**	12	14%	4	13%	1%
**CSR** ^**a**^	1	1%	1	3%	-2%
**Government**	15	17%	7	23%	-6%
**Indigenous**	4	5%	2	6%	-2%
**NFP** ^**b**^	13	15%	3	10%	5%
**Research**	12	14%	3	10%	4%
**Tourism**	14	16%	4	13%	3%
**Training**	1	1%	1	3%	-2%
**Total**	87	100%	30	100%	
**MSP**^**c**^ **and AG**^**d**^	8		3		
**Grand total**	95		33		

^**a**^Corporate social responsibility

^**b**^Non-for-profit

^c^Multi-stakeholder platform

^d^Advisory group

The sectors represented by the participants are described below.

- Aquarium–Private companies propagating and selling coral.
- Charter yacht–Chartering vessels for tourism and scientific purpose.
- Citizen science–Organisations that mobilise citizens to engage in various scientific activities such as small-scale restoration efforts, in-water and other virtual monitoring of reef ecosystem health or particular species.
- Contractor–Private contractors for marine management services such as crown-of-thorns starfish control.
- Corporate Social Responsibility (CSR)–Corporations that have programs on reef restoration through their CSR activities.
- Government–State and Commonwealth government departments and agencies involved in marine management or relevant to reef restoration in the GBR.
- Indigenous–Traditional Owners, Indigenous rangers, and Indigenous-identified persons working in or have relevance for reef conservation or restoration in the GBR.
- Non-For-Profit (NFP)–These organisations include environmental non-governmental organisations, citizen scientists, volunteer groups and community organisations with objectives tied to reef conservation, or restoration.
- Research–Researchers and/or research organisations working on reef conservation, or restoration.
- Tourism–Tourism operators and peak body in the GBR.
- Training–Trainers and/or training organisations for skills and qualifications relevant to reef conservation, or restoration.

The interviews were semi-structured, where a set of eight core questions about potential collaborators, their potential roles, motivating benefits, challenges and enablers to scaling and collaboration guided the interviews and additional impromptu questions were formulated based on participants’ responses (see S1 File for the eight core interview questions). Participants were asked to limit their responses to larval reseeding and coral propagation because these two broad categories of interventions had the highest technology readiness level relative to the other interventions examined by the RRAP. The team conducted 32 interviews remotely through Microsoft Teams and WebEx, or mobile phone, and 1 in-person interview. These interviews were recorded and transcribed. The team reviewed each transcript for completeness and correctness.

**Fig 1:**
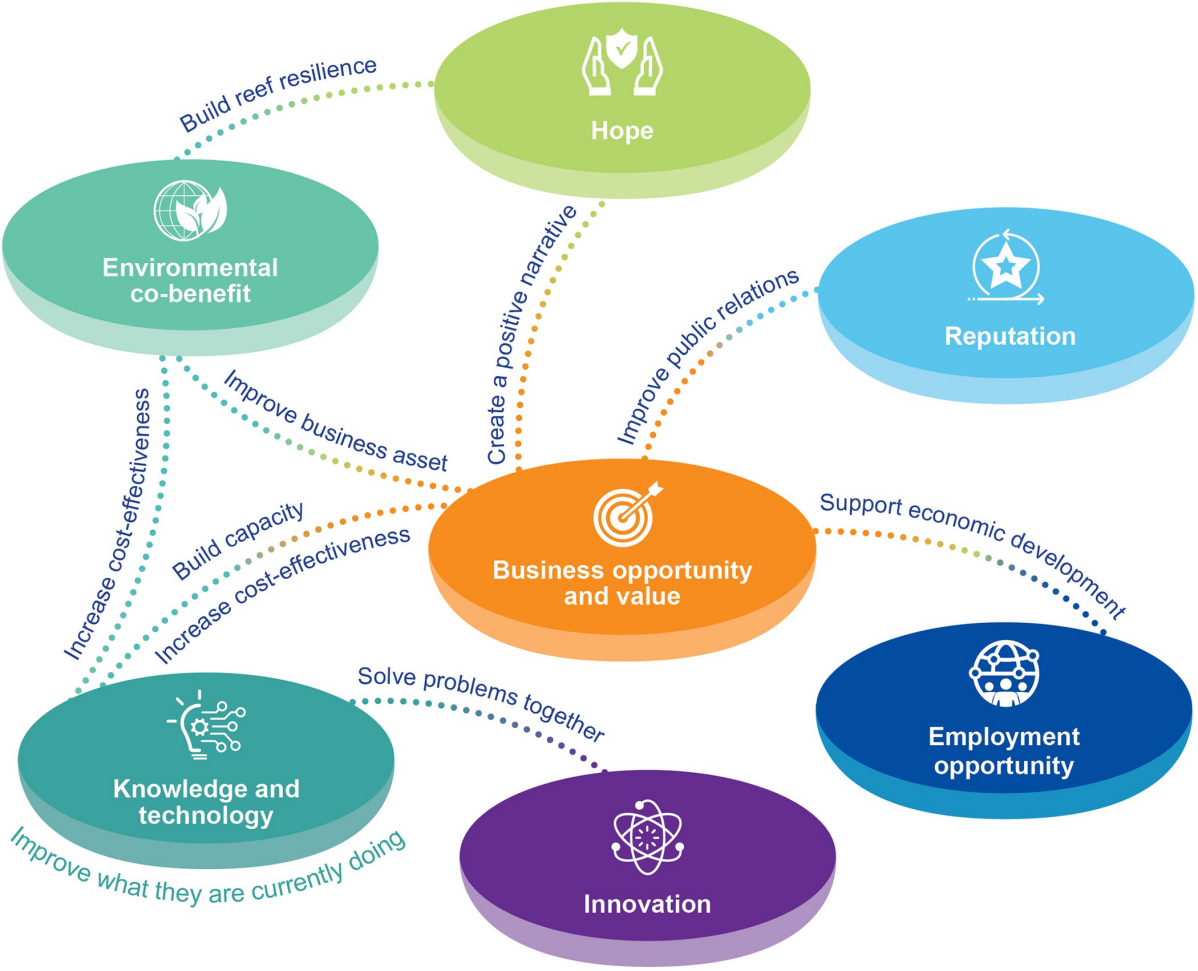
Participant-identified desirable benefits for participating in deployment of moving corals or coral aquaculture.

The team used the interview questions to identify initial themes for coding the transcript in NVivo 12.0 (QSR International). The initial themes were potential collaborators, roles and benefits. New codes were developed inductively from emerging themes such as competitive advantage. The four types of capital, namely physical, human, organisational and financial, were used to categorise each competitive advantage. The scaling strategies developed by Islam [44] were used to categorise identified roles. When coding benefits that motivate collaboration, connections between benefits emerged, and these connections were represented in Fig 1 and elaborated in Section 4.3.

### 3.3 Limitations

The first limitation relates to the participation of Traditional Owner groups. Indigenous Australians, particularly Traditional Owners, who have recognised customary connections to identified areas of the GBR, are essential potential collaborators for coral restoration efforts. Our study into CSBM is one of several participatory social research activities under the Stakeholder and Traditional Owner Engagement Sub-program of RRAP. A sister project under that program of work is a dedicated, co-designed study to identify biocultural risks and opportunities with Traditional Owner groups from future restoration interventions, including identifying aspirations for leadership and partnership of those activities. This study was intentionally designed to have limited Traditional Owner participation to minimise participant fatigue. Traditional Owners’ perspectives were captured in the project reports [62, 63] of the sister project. We were asked by the report authors not to present or discuss their findings in this paper because these reports have not been published. The findings from the sister project are complementary to this paper even though they did not use a CSBM lens to design their research.

The second limitation relates to the participation of funders. Funders are often seen as one of the collaborators in cross-sectoral collaboration in coral restoration. At the time this study was undertaken, parallel, targeted research was being planned within the RRAP to scope and investigate potential funding mechanisms to support scaling of restoration and adaptation interventions through biodiversity credit markets. That research at the time of writing is still underway. We want to, however, highlight that the government, tourism and Corporate Social Responsibility (CSR) sectors among our participants are common funders [20]. While we did not ask them about funding mechanisms in the interviews, the benefits that motivate them to collaborate were included in the interview questions. We have also tried to address this limitation by discussing literature on funders’ perspective in Section 5.4.

The third limitation relates to the snowballing method. Sectors such as large environmental engineering firms, or consulting companies that provide project management and large-scale project development could be potential collaborators, but the project team did not get referrals for these sectors. The snowballing method can be biased by the referral preferences of the initial contacts and their referrals. Despite this limitation, snowballing is a widely accepted sampling method for social research, and the project team continued to ask participants for referrals until a saturation point was reached–i.e., the referrals were repeating.

A fourth potential limitation relates to the adoption of a case study approach and the broader transferability of findings. We decided to use a case study because it allows us to examine the particularities and rich details by focusing on a specific place-based and programmatic context. There are limitations with drawing generalisations from case studies, but we avoid generalising categories of collaborators, roles and benefits with the results because these categories would probably vary in different contexts. However, in developing and applying the analytical framework to elicit those roles and benefits, we argue that the higher order findings are widely transferable to other contexts, as is the method of analysis to ‘like’ problems in these other contexts.

## 4 Results

### 4.1 Potential collaborators

Participants identified potential future collaborators across 10 sectors for scaling up larval reseeding and coral propagation interventions in the GBR (Table 2). Many of these future collaborators already operate in the GBR space, though not in reef restoration. This is reflected in their competitive advantages, which include existing infrastructure, networks, workforce, and know-how that are transferable to reef restoration. All potential collaborators possess at least two types of resources, with human resources being the most common (Table 2).

**Table 2:** Potential future collaborators, their competitive advantages, and types of resources.

Potential collaborators	Competitive advantage identified by participants	P^a^	H^a^	O^a^	F^a^
**Aquarium operators**	Some of their existing aquarium facilities potentially can be converted or expanded for coral aquaculture. This lowers capital investment in facility development. They have the know-how for propagating and caring for corals and operating aquarium facility. Some have licence to harvest corals, local reef knowledge and diving licence.	X	X		
**Charter boat operators**	These operators have vessels, and some have equipment such as holding tanks and crane, etc. They have the capability to operate vessels, navigate and operate in marine environment.	X	X		
**Commercial fisheries**	Specifically referring to fisheries for aquarium industry, tropical rock lobster and sea cucumber. They have vessels and equipment such as holding tanks and crane, as well as capability to operate vessels, dive and work in marine environments.	X	X		
**Consulting companies**	International and national large consulting firms with experience in managing large-scale projects and risks associated with project delivery. They often have inhouse multi- or transdisciplinary expertise and existing networks of sub-contractors.	X	X	X	
**Marine contractors**	They either have their own or have existing connection to those who have vessels and on-boat equipment. They have divers and some of which may be certified as commercial divers as part of their workforce, and standard operating procedures. They have the capability to operate vessels and work in marine environments.	X	X	X	
**Non-for-profit (NFP) organisations**	Some NFPs have their own funding and can co-invest. Their volunteers’ labour is free, and some NFPs have significant number of volunteers and local reef knowledge. They have experience with raising funds from the private and philanthropic sectors, as well as mobilising the public or communities to take actions. They have existing networks with industry, other community-based organisations and government.		X	X	
**Tourism operators**	They have their own vessels and regularly travel to the GBR. As a sector, they have a large footprint and extensive networks throughout the GBR. They have the capability to operate vessels and work in marine environments, as well as local reef knowledge and divers in their workforce.	X	X	X	
**Traditional Owner entities**	Traditional Owners hold rights to many parts of the GBR and traditional ecological knowledge. Some Traditional Owner groups have ranger programs and Traditional Use of Marine Resources Agreements–both of these come with funding for environmental management. Some groups are already active in reef management and have local knowledge.		X	X	
**Research institutions**	Their strength is in research and development of science and technology. They add creditability to data collection or assessments and provide decision-support.		X	X	
**Government entities**	These are Commonwealth and state government organisations that are already working and investing in marine management in the GBR. Some of these entities regulate coral restoration.	X	X		X

^a^The letters are abbreviations for resource types described in Section 2.1: physical, human, organisational, and financial.

As potential funders are not included in the project scope, it was not expected that these collaborators to provide financial resources. Co-investment of physical, human and organisational resources was not considered a financial resource.

### 4.2 Collaborators’ potential roles

Participants described nine potential roles that these future collaborators could play in scaling up larval reseeding and coral propagation interventions in the GBR (Table 3). Five of these roles are not involved in the “doing” of coral restoration but are associated with the system structure for resource allocation and mobilisation. This pattern was reflected in the scaling strategies that these roles were mapped to, as most of them were related to ecosystem growth strategies (see Section 2.2) that focus on growing or sustaining a supportive business ecosystem, as opposed to organisational growth strategies that focus on growing organisational size.

**Table 3:** Potential roles identified that collaborators could play in scaling up future coral restoration mapped to the types of organisational or ecosystem growth scaling strategies.

Role	Description	Organisation^a^	Ecosystem^a^
**Adopter**	Adopt the coral restoration techniques developed by RRAP to restore reefs for their own coral projects or business activities	EG	
**Adviser**	Provide technical or scientific guidance and advisory inputs to others such as the implementation and management entities in a program		TA, L
**Advocate for investment**	Advocate for resource support including funding and in-kind co-investment		A
**Capacity building/ back-office support**	Provide back-office support such as financial and contract management, and grant and report writing. Capacity building such as coaching/mentoring on project management, leadership and technical training (e.g. reef restoration techniques, coxswain tickets, diving).		TA
**Connector**	Connect a program with networks, Traditional Owners and stakeholder groups–e.g. reef researchers, implementers, industry players and community organisations–to help share information, communicate local inputs and find collaboration opportunities.		C
**Citizen/ community mobiliser**	Mobilise the public and citizen scientists into actions for a cause or interest as it relates to scaling up larval reseeding and coral propagation interventions.		A
**Deployer/ implementer**	Deploy or implement a part or all of the larval reseeding and coral propagation interventions including data management, monitoring and evaluation and reporting.	EP/S	
**Project management** ^**b**^	Manage the implementation of a coral restoration project include managing sub-contractors delivering to a contract and infrastructure.	EP/S	
**Research and Development**	Undertake research to continuously improve existing knowledge and technology, and innovate new knowledge and technology for reef restoration and adaptation		RD

^a^The letters in these columns are abbreviations of organisational and ecosystem growth scaling strategies described in Section 2.2: product/service expansion (EP/S), geographic expansion (EG), advocacy (A), coalition (C), training and advisory (TA), legitimacy (L) and research and dissemination (RD).

^b^The project management role entails sub-contracting the work to other organisations.

Some potential collaborators have the capacity to play multiple roles–e.g. a marine contractor can be an adviser in one project, an implementer in another project, and a project manager on a third project.

### 4.3 Benefits motivating collaboration

Participants described seven types of benefits that would motivate them to collaborate in scaling up larval reseeding and coral propagation interventions in the GBR in the future: environmental benefit, business opportunity and value, employment opportunity, knowledge and technology, innovation, hope and reputational benefit. Most participants described these benefits not in a mutually exclusive manner, but rather as co-existing or mutually beneficial, as illustrated by the connecting lines and associated description in Fig 1. These benefits are described in detail below.

#### Environmental benefit

The highest number of participants (N = 10) identified environmental benefit as a motivator to collaborate, and it was mainly described as improving reef health, producing conservation outcomes, protecting the reef and “having a vibrant healthy resource” (Participant 5). Participant 15 indicated that his company is only interested in working with organisations and projects that have a genuine interest in conserving the environment:

*We*’*ve worked with a couple of companies we’ve chosen not to work with further because their heart’s not in the right place, they’re just trying to find shortcuts and do things sometimes dangerously for personnel or certainly not about conserving the environment, and if they can find a shortcut to do something that hasn’t got a good sustainable environmental outcome that’s not someone we want to work with.*

Participants did not see environmental and business benefits as mutually exclusive of each other. In fact, environmental benefit is seen as being parallel to or supporting business interests. Marine operators discussed the benefits of doing “something good for the environment” (Participant 29) while making a profit. Multiple tourism operators indicated that reef health is the underlying asset of their business, and their conservation interests stem from their love for the reef and their business interests. Furthermore, their environmental interests extend to the broader reef ecosystem instead of specific reef sites allocated to individual operators, as Participant 26 explained:

*So we have an appetite to be able to understand our sites and look after them and cherish them. Not only for our own reasoning, but also, we understand there’s no point just protecting our little patches if the whole ecosystem is not doing well because i*t*’ll just–it’ll still fall over so we know it’s bigger.*

#### Business opportunity and value

Participants described this benefit as opportunities for creating new or supporting existing businesses through improving profit or increasing the value of a business’ service or product. For example, reef restoration can improve the quality of marine tourism sites–the sector called these high-value reefs–and, consequently, their visitors’ experience. Two participants from the tourism sector added that collaborating on reef restoration can also create marketing value for their business–e.g., such collaboration can help to demonstrate that they are ecologically conscious operators. Participant 26 explained: “It’s our way of significantly demonstrating to the world that if they are on our vessels and see work that we’re part of, and we can advertise and market it and this and that, the industry goes, oh tourism is actually playing its role”. Another tourism operator (Participant 8) explained that such a partnership could potentially help them develop a new type of experience for visitors who want to get involved in reef restoration:

*So this idea of regenerative tourism, some people call it voluntourism, that’s the future. Ecotourism as we know it, if you’re doing that old model, you’re not going to succeed, you’re not going to thrive. So that’s what* we’re *looking for, is we’re looking to appeal to those people who want to make a difference and not just do it in a superficial way.*

A participant from the aquarium industry sees collaborating in the future scale-up of coral propagation as an opportunity for building resilience into their business because it can diversify their income stream.

#### Employment opportunity

While only three participants discussed employment opportunity as a motivating benefit, they highlighted who should be the beneficiary. One participant (Participant 19) pointed out the importance of creating employment opportunity for local reef communities instead of bringing in a workforce from elsewhere:

*So an example of that for state government was main roads where they basically made sure a percentage of the contract had to be local procurement rather than Indigenous, because what would happen is you might have a contractor and they bring people up for the season, and they live in road camps and fly out every month and all of the money is going back out. So how do you have a policy to actually make sure there’s a difference from that spend in the local community?*

Another participant saw that the employment opportunity could be a training ground for new marine sector graduates to gain professional experience and prepare for "the next bigger job that they might go onto" (Participant 29).

#### Knowledge and technology

The seven participants who identified gaining knowledge and know-how or using new technology as a motivating benefit are engaged in activities related to reef restoration–e.g., rehabilitation of ship grounding sites and restoring reefs with high tourism values. They believe that by collaborating with research programs such as RRAP to scale up reef restoration, they will be able to gain knowledge and technology to improve what they are currently doing, as explained by Participant 2:

*Because, obviously, collective action through tourism means that the tourism industry wants to focus on retaining and rebuilding the health of their sites…* *The science informs how to plant better and more cost-effectively which means the operators can plant more. Which means because we have more scale we have more data to better-inform how to grow corals better.*

Some participants saw the benefits of using the knowledge and technology they can gain from future collaboration with a research program to scale up reef restoration to build the capacity of their employees and businesses. A participant from the tourism sector gave an example of building their staff’s knowledge to improve how they communicate to tourists about marine conservation efforts.

#### Innovating together

Three participants see themselves as innovators, and collaborating on the scaling up of reef restoration presents an opportunity for them to innovate with scientists. Participant 15 explained his interest in working with scientists to solve problems as a valuable benefit:

*… one of the things that I’ve talked to a number of researchers involved in this area, is how do we build this and scale it up and how do we take it to the next level? And that’s one thing that I’m very keen to do, in fact I love having a challenge and coming up with new solutions on how to roll something out and how to expand something.*

#### Hope

Three participants explained that collaborating on reef restoration gives them hope in the face of a daunting global challenge posed by climate change. Participant 5 described how his perspective about the future of the GBR has become more positive since being involved in reef restoration:

*It’s just, you know, when you’re trying to explain to somebody what climate change will mean to the Great Barrier Reef, it’s a downward spiral, whereas now that we’re involved in restoration work, we can end that discussion with a little bit of hope and say well, look, we’re involved in that work and you can even point it out to them, so they can go and have a look at it and say that may be increasing the resilience of the site to any future impacts through coral bleaching, et ceter*a.

#### Reputational benefit

Two participants talked about the importance of reputation to their businesses and saw reputation as a valuable benefit to motivate their collaboration. One Participant described their organisation as a conservation- and research-based agency, and they need to partner on actions aligning with their organisational reputation. Participant 8 described reputation at a sectoral scale, "We’d like to see this region being benchmarked by the rest of the world". Partnering to restore the GBR is to protect the reputation of the tourism sector.

## 5 Discussion

### 5.1 Diversity of collaborators and roles

Our findings present a diverse landscape of potential collaborators and the roles that they can play to contribute to scaling coral restoration. The potential collaborators include organisations from the public, private and nonprofit sectors. While many of these potential collaborators are already involved in coral restoration in the GBR, we identified potential new collaborators, namely aquarium operators and commercial fisheries. Our findings resonate with the diverse range of actors involved in coral restoration efforts already documented in the literature–e.g., tourism operators [40], government, private sector, universities, non-governmental organisations, community, dive clubs, students [41], and citizen scientists [39]. Diverse combinations of potential collaborators and resources can contribute to scaling larval reseeding and coral propagation in different ways because of the distinctive characteristics of these interventions. Under existing, available methods larval reseeding is associated with spawning events and the intervention only lasts several days, while coral propagation involves year-round efforts to collect, rear and transplant coral fragments. Having access to a large number of appropriate vessels in as many regions of the GBR as possible is key to scaling larval reseeding. It is not necessary to own these vessels as they are only needed for a few days. Scaling coral propagation requires dedicated onshore facilities to rear the coral fragments, more sophisticated logistics to transport them safely to target reefs, and divers to transplant them. Different collaborators and resources are needed for these coral propagation activities. Some studies examined the relationship between diversity and business model innovation [e.g., 64, 65], but whether diversity in collaborators and their roles contribute toward innovation in scalable CSBMs for coral restoration has not been examined. Future research could, for example, look at the mechanisms by which diverse collaborators and roles can scale up value creation, delivery and capture associated with coral restoration.

### 5.2 A typology relating to ecosystem growth strategies

Resources associated with the potential collaborators are well distributed among physical, human and organisational capital, and most collaborators can provide two types of resources, either physical and human, or human and organisational. Financial capital was not identified as a resource discussed by the potential collaborators in this study, which is not surprising as we did not target funders as key participant category, but we discuss some of the potential alignment with funders in Section 5.4. Organisational capital, such as networks to other potential collaborators and access to government funding, supports ecosystem growth strategies, including coalition building, advocacy work, and knowledge dissemination. Our case study participants perceived potentially playing a diverse range roles to scale up coral restoration. Some of these roles have previously been identified in the literature [e.g., 39–41, 66], but the roles of advisor, investment advocate, capacity building/back-office support, and connector are novel. For examples, some participants indicated that they could help scale coral restoration by lobbying for more public funding, while others said that they could provide training to future restoration practitioners and connections to other collaborators. Ecosystem growth strategies scale up desirable impacts by improving the environment in which a CSBM operates. Studies pointed out the importance of an enabling environment for scaling restoration. For example, McAfee et al. [67] attributed the funding of Australia’s shellfish reef restoration to the initiative proponents’ ability to galvanise public and industry support by aligning project goals with their interests. Mansourian [68] identified governance and economic factors as motivators that initiate scaling-up forest restoration, enabling its implementation at scale, and are key to sustaining these efforts in 10 regions. Roles relating to ecosystem growth strategies are not well captured in collaborative business model typologies such as those by Ciulli et al. [36] and by De Man and Luvison [35] because those typologies focus on the individual organisation, and not interactions between an organisation and environment. As these interactions can be an essential part of why organisations collaborate in CSBMs, future research could develop a typology of CSBMs based on roles, particularly those relating to ecosystem growth strategies.

### 5.3 Capturing non-market values

**Fig 2:**
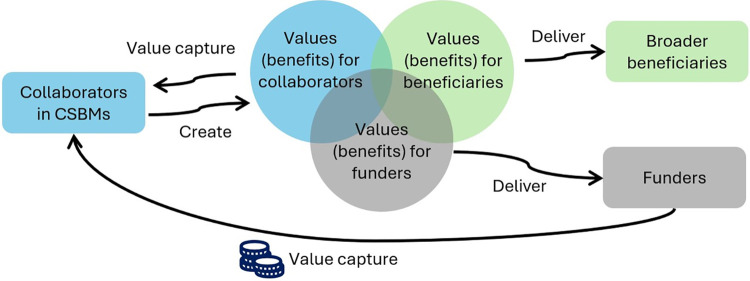
A schematic clarifying the relationship between values (referred to as benefits in this study) that are primary interest of collaborators, funders and broader beneficiaries.

Six of the seven identified benefits–i.e., hope, innovation, knowledge and technology, reputation, employment, and environmental benefits–that motivate collaboration have non-market values because these values are not reflected in market prices. There are non-market valuation methods to estimate the value people place on ecosystem goods and services for which there are no market prices [e.g., 66], but it is more challenging to put a monetary value on benefits such as hope, innovation and knowledge. In a conventional business model, benefits generated by a business for its customers are referred to as value propositions and are embedded in its marketable goods and services. CSBMs create multiple bundles of benefits or value propositions for funders, broader beneficiaries, and collaborators (Fig 2). Value propositions that are not traded in markets require considerations for how to capture or attribute the value associated with these benefits back to the creator of these value propositions. There are examples of markets and market mechanisms such as forest tourism, optimised entry charges, debt-for-nature swaps, transferable development rights, and carbon markets used to capture non-market values [69]. The non-market nature of the many benefits or value propositions that motivate collaboration implies that there is a need to embed alternative mechanisms enabling collaborators to capture these values in the CSBMs. These mechanisms could include sharing arrangements, for examples with knowledge and technology, and marketing arrangements to build reputation.

Not all collaborators in a CSBM will be interested in the same bundle of values, as our interview participants indicated that different benefits motivate them to collaborate. Considerations are also needed to address potential tensions arising from the different interests in these values (the blue bubble in Fig 2) and trade-offs between values for funders (the grey bubble), broader beneficiaries (the green bubble), and collaborators. Oskam et al. [70] explored the tension of mutual value versus individual value and proposed "valuing value" as a solution, where collaborators search for a result that satisfies all actors. They identified two patterns of valuing value: collective orchestration (i.e., actors have a clear mutual vision and time horizon with integrated sustainability goals) and continuous search (i.e., actors focus on environmental and social value creation and search for economic viability). Similar to Oskam et al.’s [70] study, we did not examine the influence of power or unequal relationships, which can emerge in cross-sectoral collaborations. In collaborative business model literature, terms such as alliances, partnerships, and collaborations do not imply a normatively desirable arrangement or equal relationship [e.g., 26, 35]. As power imbalances invariably influence decisions, such as those around how resources and captured values are shared, the dynamic of power warrants more attention on how it affects the design, desirability, and sustainability of CSBMs.

### 5.4 Aligning values with potential new funders

Some of the benefits that motivate collaboration identified in this study could speak to the objectives of emerging funders of coral reef restoration to harness new funding streams from the private sector. While primary funders of terrestrial and marine restoration were historically governments and development banks (e.g., World Bank, Global Environmental Facility), the market for marine and terrestrial restoration is maturing and there has been growth in sustainable financing instruments and private-sector investments [20]. For example, the Global Fund for Coral Reefs (http://globalfundcoralreefs.org) is the first United Nations Fund dedicated to marine restoration, targeting coral reef-positive business models that generate positive environmental, social and economic benefits [71]. Opportunity also exists for coral restoration business models to leverage funding sources that value the importance of coral reefs in supporting tourism, fisheries, and biotechnology industries–all key elements of the Blue Economy [71, 72]. Reef restoration has been reported to attract a higher volume of private sector funding than traditional forms of reef management–potentially driven by an interest in improving coral health at high-value tourism sites and enhancing the tourism experience either by participating in restoration tourism or by seeing patches of a healthy GBR [66]. Private sector investors such as Qantas Airlines, Australia’s largest airline, have committed AUD 10 million investment to accelerate the restoration and adaptation of the GBR [73]. Shared values that align funders and collaborators could drive initial and ongoing participation to collaborate and funding support.

## 6 Conclusion

Active coral reef restoration has wide support as a management strategy to address the decline of the coral reef systems worldwide. However, the gap between the scale of current coral restoration practices and what is needed to match the rate of degradation is glaring. As a complex problem, scaling requires funders and practitioners alike to address fundamental technological, operational, informational and socio-institutional dimensions of scaling. The search for solutions includes developing business models to sustain and scale restoration efforts by several order of magnitude. The collaborative character of these models is critically important as any new set of interventions does not land into an empty seascape, but into an existing and diverse assemblage of actors, interests, capabilities and motivations. Moreover, the scale and complexity of the challenge will require effective networking and collaboration across different types of actors, and encourage two-way flows of knowledge and other benefits between local and extra-local initiatives.

This study expands our understanding of the diversity in potential collaborators, their resources and roles in relation to scaling strategies and benefits that not only motivate collaboration but may attract new funders. The characterisation of potential roles, by prospective collaborators, is particularly rich and extends well beyond direct involvement in implementing restoration interventions. Many of the roles relate to ecosystem growth strategies (e.g., advisor, investment advocate, capacity building/back-office support, and connector) are novel from existing literature and reflect participants’ diverse perspectives of how to scale coral restoration by influencing the business environment. Our findings can contribute to innovating scalable CSBMs because they inform the articulation of strategic business partners, resources, activities and value proposition–all are key elements in a business model. The suitability and success of these new business models will need to manage tensions that may arise from the different interests in and trade-offs between diverse collaborators, funders and the broader beneficiaries. Similarly, these new models must take into consideration how power imbalances influence decisions about how resources and captured values or benefits are shared amongst CSBM collaborators. Equally important to consider is how our findings may apply to coral restoration efforts in different jurisdictions is local context, including key players, governance structure, resources and culture, because local context influences what is feasible and desirable in CSBMs. We also identified knowledge gaps that require future research such as mechanisms for capturing non-market values to collaborators and a typology of CSBMs that help us to understand the interaction between collaborators and the business environment. This study highlights opportunities for alternative pathways to scaling business models for coral restoration beyond reducing unit cost and increasing funding.

## Supporting information

S1 File Interview questions. (DOCX)
