# Supplementary material for: Why partner? Harnessing value from collaborative sustainable business models to restore coral reefs at scale
Source: PLoS One. 2024 Dec 16;19(12):e0315094. doi: 10.1371/journal.pone.0315094 (PMC11649128; doi:10.1371/journal.pone.0315094)
Supplement: S1 File — (DOCX) [file pone.0315094.s001.docx]

**Why partner? Harnessing value from collaborative sustainable business models to restore coral reefs at scale**

**Supporting information: S1 Interview questions**

Opening questions – act as warm-up/build rapport and help interviewer to get a sense of the participant’s perception about reef restoration, and specifically larval reseeding and coral propagation.

1. Can you talk about your role in XYZ organisation/project? (Potential follow-up if not already mentioned in the response – What does your organisation do? Where does your organisation work?)
2. What is your perception about reef restoration? (Then move to perception about larval reseeding and coral propagation, including their interests and concerns. Depending on the response, gradually guiding the discussion toward perception about these interventions at scale. This helps bound the discussion below.)

Core interview questions

1. Would your organisation be interested to be involved in these kinds of reef restoration a few years into the future? (Prompt discussion around reasons for why/why not. Linking reasons to discussion around benefits - what would motivate your organisation to be involved in these interventions? The discussion around reasons may also lead to concerns.)
2. In what ways would your organisation be involved? (Prompt discussion around roles, capability and partnership arrangement/model.)
3. Who else do you think could also play a role in these kinds of reef restoration? What roles do you think they could play?
4. Who could play a coordinating role between funders, researchers and implementers? (Only prompt this question if the roles discussed do not include coordinating between funders, researchers and implementers. Note that they can be different for each intervention. This helps to identify *in situ* knowledge brokers.)
5. What do you see as potential barriers or challenges to organisations partnering to implement these interventions?
6. What would enable these interventions to get implemented in the Great Barrier Reef at a large-scale?
